# Stealing complex network attack detection method considering security situation awareness

**DOI:** 10.1371/journal.pone.0298555

**Published:** 2024-03-21

**Authors:** Bo Xi, Huiying Liu, Botao Hou, Ying Wang, Yuling Guo

**Affiliations:** State Grid Hebei Electric Power Research Institute, Shijiazhuang, Hebei, China; Sunway University, MALAYSIA

## Abstract

Tracking and detection have brought great challenges to network security. Therefore, this paper proposes a monitoring method of stealthy complex network attacks considering security situation awareness. By constructing a tracking model of invisible complex network attacks, public monitoring nodes are selected for monitoring. The cost of a single monitoring node is calculated by the algorithm, and the monitoring node is determined by the monitoring node algorithm, so as to reduce the resource occupancy rate of the monitoring node and improve the monitoring accuracy. The simulation results show that this method is stable in the range of 1000 to 4000 nodes, and can effectively monitor the complex network attacks of stealing secrets.

## 1 Introduction

With the rapid development of network technology, complex network has become an important part of modern society, involving many fields such as finance, military and politics. However, the fragility of the network makes it vulnerable to various attacks, among which the stealing attack is a common network threat. Therefore, it is of great significance to study a monitoring method for stealing complex network attacks. Reference [1] proposed DNS (Domain Name Server) protection to prevent cheating and poisoning attacks, and used asymmetric passwords to encrypt important information in messages to prevent messages from being tampered with, thus preventing the domain name system from being attacked by cheating and cache poisoning. However, this method needs a relatively long calculation time, which may slow down the response speed of DNS system. Reference [2] proposed an enhanced state protocol based on registered ARP (Address Resolution Protocol) requests, which defeated cache poisoning attacks by discarding unregistered replies, enhanced the address resolution protocol into a stateful protocol, and sent requests according to the number of replies received from fake attackers. However, in order to verify the registration status of each reply, the network device needs to maintain a status table, which may increase additional storage and management overhead. Reference [3] proposed an encryption algorithm based on WSN (Wireless Sensor Networks) to reduce power consumption, and used asymmetric password RSA to set different key sizes for data blocks to complete information encryption and decryption. However, this method needs to consider the actual capabilities and limitations of nodes. Reference [4] proposed the protection of DNS infrastructure based on Enc-DNS-HTTP (Encryption-Domain Name Server-Hypertext Transfer Protocol). The public key is transmitted through the secure communication between the client and the DNS server, and the client-server communication is encrypted through the key, thus achieving the purpose of resisting the HTTPS (Hypertext Transfer Protocol Secure) attack. However, the public key transmission of this method may be intercepted or tampered by attackers, which may easily lead to communication insecurity. Reference [5] proposed a network attack detection method based on adaptive immune computing. The density-based clustering algorithm was used to preprocess the self-training data, and the clustering analysis was carried out to remove the noise and generate a detector to complete the anomaly detection. However, this method is greatly influenced by the amount of network data. Reference [6] proposed a DDoS (Distributed Denial of Service) attack detection method for OpenFlow network based on Sibson distance, which used multi-agent controllers to reduce the load of the main controller, designed a layered DDoS attack detection architecture, and completed network attack detection. However, the resource occupancy rate of this method is low. Reference [7] proposed heterogeneous data processing and network attack detection based on a two-level segmentation model, which integrates heterogeneous data, establishes a network attack model based on the analysis of network attacks of heterogeneous data, and completes heterogeneous data processing and network attack detection. However, the attack detection accuracy of this method needs to be verified. Reference [8] proposed an optimization method based on semi-supervised learning to detect wireless network attacks. According to the feature vector and the original feature weight vector, the characteristic value of cluster network traffic was calculated, and the prediction result of network attacks was obtained by using the feature weight vector to complete wireless network attack detection. However, this method has high computational overhead in the retrieval process. Reference [9] put forward an attack detection algorithm for smart grid security stealth network. On the basis of collecting data transmitted from sensors to automatic generation controllers by telecommunication links, Kalman filters and detectors are used to obtain hidden attacks. However, the attack range detected by this method needs to be verified. Reference [10] proposed SQLI (Structured Query Language Injection) suppression based on encrypted query and secure matching. Use a separate key to encrypt each user’s information in the database, and compare the encrypted user name and user entries in the database to complete user authentication. However, this method is easy to generate data calculation redundancy. Reference [11] put forward a packet replay prevention protocol for secure B5G (Beyond 5G) networks, and built a Dolev-Yao model by using the protocols of message authentication code, symmetric cipher and elliptic curve cipher to improve the attack detection effect. However, the training time of this method model is long.

In the existing research, the monitoring methods for complex network attacks often ignore the importance of security situation awareness. Security situational awareness can warn and discover potential attacks in advance, thus improving the defense capability of the network. Therefore, this paper proposes a method to monitor the secret-stealing complex network attacks considering security situation awareness. This paper analyzes the characteristics and behavior patterns of secret-stealing complex network attacks. By constructing the tracking model of invisible complex network attacks, the public monitoring nodes are selected and the cost is calculated to determine the monitoring nodes, so as to realize sensitive monitoring of complex network attacks. The contributions of this method are as follows:

1. Considering the importance of security situational awareness, a monitoring model based on security situational awareness is constructed, which can effectively find and track the secret-stealing attacks.
2. A public monitoring node selection algorithm is designed, which can effectively cover the whole network at the minimum cost and improve the monitoring effect.

## 2 Stealing complex network attack monitoring considering security situation awareness

### 2.1 Tracing to the source of stealing complex network attack

Traceability stealing complex network attack means that the security must be changed from network to data, and it is a kind of network security protection mode with data security as the center. Traceability of complex network attacks includes many aspects: establishing traceability system; traceability based on adjacent nodes; tamper-proofing or forgery of complex network packets in the process of traceability; optimization of dynamic probabilistic packet marking for traceability of DDoS attacks. Network traceability on SDN (Software-defined Networking) networks; coding characteristics, evolution characteristics and functions of malicious codes in the traceability stage; traceability dynamic evaluation model of complex network attacks based on AHP (Analytic Hierarchy Process); detection of attack paths of complex network nodes; research on threat traceability methods based on IDS alarms and rootkit, etc.

**Fig 1:**
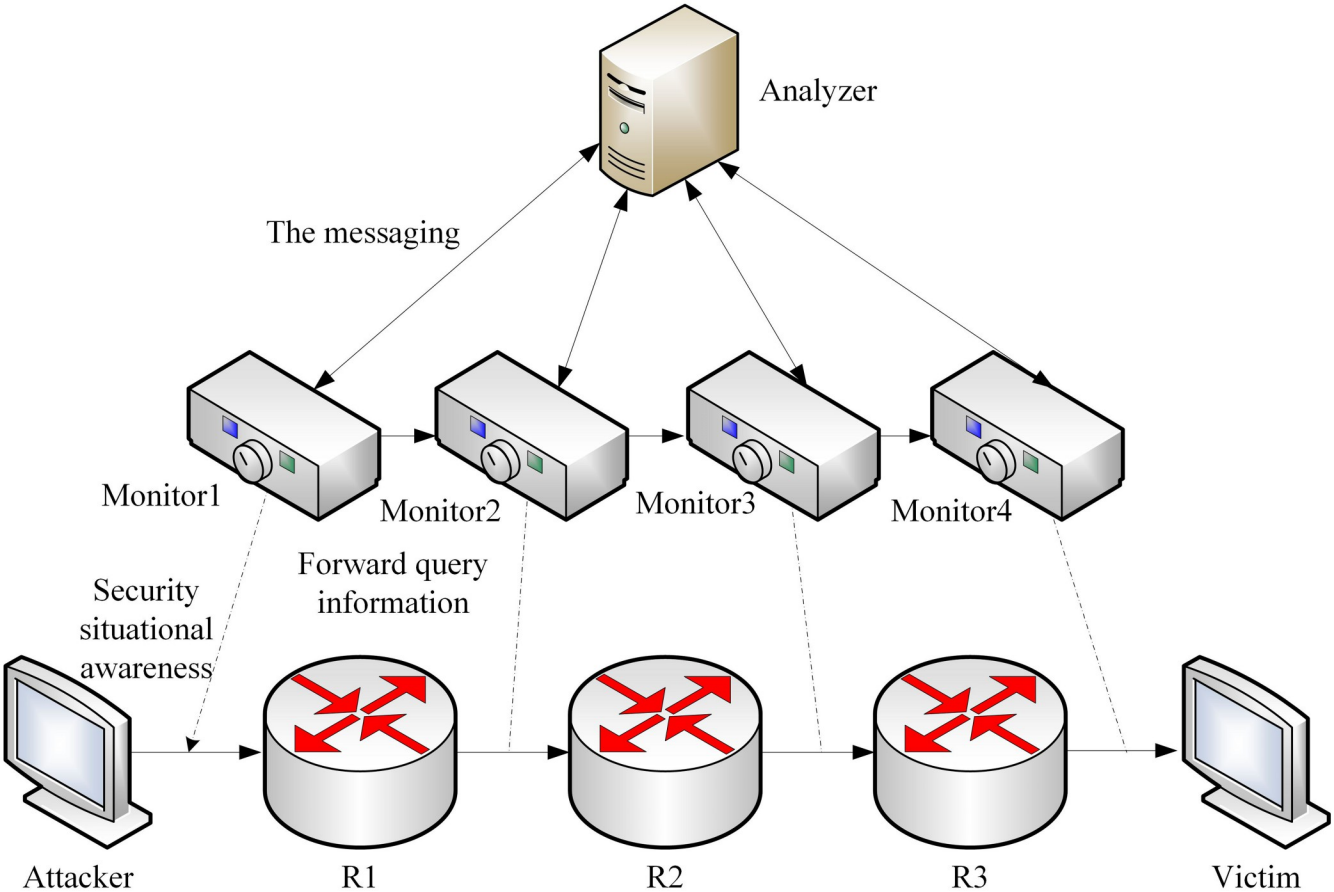
Traceability model of stealing complex network attack.

In order to improve the ability of monitoring the stealthy complex network attack, the traceability model of the stealthy complex network attack is constructed firstly. In order to realize the detection, understanding and projection of the security situation of the stealthy complex network, a network monitor is set on the router of the shared stealthy complex network and the edge entrance, and the security situation of the datagram is perceived, understood and projected, so as to achieve the goal of tracing the source of network attack. Distributed intrusion detection technology is a technology that uses multiple intrusion detection systems deployed everywhere in the network to work together. Each monitoring point can use its own intrusion detection system and share and coordinate the detection results with other monitoring points. Each network monitor will collect and analyze network traffic and activity data in real time. The theft complex network monitor installed on the edge entrance router is mainly responsible for the detection of datagrams, which may include network traffic, transmission protocol, application behavior and so on. By integrating data through distributed technology, a global view of the whole shared network is formed and joint decisions are made, so that the network monitoring system can respond to security threats more quickly and flexibly and realize security situation awareness. When a network attack datagram is detected, a stealthy complex network monitor sends attack information to the analyzer to reconstruct the inter-domain path [12]. The traceability model for stealing complex network attacks is built as shown in Fig 1.

In Fig 1, the execution steps of a stealthy complex network monitor are as follows: When an attack occurs in a stealthy complex network, the most recently distributed network monitor initiates a query path request and traverses all network monitors. When the corresponding response information of the path query is sent back, the stealthy complex network monitor constructs the attack path based on the response information for tracing the source of the attack. Data is created by producers on a complex stealing network and flows to different locations in different ways. When the data flow occurs, the user modifies and edits the data to form a new data. Therefore, in the stealing complex network system, there is a strong correlation between the data. The information contained in each data is unique, the owner attribute is different, and the creation time is different. Therefore, the electronic fingerprint information can be extracted by extracting the related features. The system will generate a unique electronic fingerprint for the data to be transmitted. Electronic fingerprint is usually a fixed-length string calculated by using encrypted hash function according to the content of data. This electronic fingerprint can be regarded as the unique identifier of the data. Transmitting data carrying electronic fingerprints to a target system or a target device. In the transmission process, the electronic fingerprint information can be transmitted together with the data, or it can be transmitted separately and re-associated at the target. The receiving system will receive the transmitted data and monitor the data. By identifying the electronic fingerprint in the data, the system can confirm the integrity and authenticity of the data, and detect whether it has been tampered with or unauthorized access, and record all the nodes through which the data flows, the processing steps and the verification results of the electronic fingerprint. These records can be used to track the source, transmission path and processing of data. Therefore, the data flow is controlled by electronic fingerprint tracking of data, which provides a basis for the reconstruction and tracking of the attack path of stealing complex networks.

When unauthorized access, malware attacks, data leakage, illegal access attempts and other forms of behavior occur in the invisible complex network, once the host invisible complex network monitor detects these abnormal behaviors, it will send a request message to the analyzer to determine whether it is necessary to further track and analyze the potential attack path across regions, so as to improve the ability to detect and respond to the attack behavior in time. After receiving the request information, the analyzer compares it with the alarm information sent by the stealing complex network monitor deployed on the edge entry router. When the two match successfully, it is verified that the stealthy complex network attack is a cross-region attack [13]. In this case, the corresponding query request for the attack path shall be sent to the analyzer deployed in other stealthy complex network regions, and the query attack path instructions in the domain shall be traced until the attack source of the attack is successfully traced [14].

### 2.2 Monitor node selection

#### 2.2.1 Common node selection

Compute the cost of monitoring nodes and select monitoring nodes by selecting monitoring nodes algorithm for stealing complex network attacks [15]. In the stealing complex network, the network topology connection is firstly analyzed, which is the carrier of the monitoring node of network attack, namely:

$$G=\left(V,E\right)$$ (1)

In the formula, *G* represents the complex network topology connection of stealthy type; *V* represents the set of all nodes in the stealth-type complex network; *E* represents the corresponding set of all direct links in a stealth-type complex network.

The adjacency matrix is fused with the topological connection of the stealthy complex network, where the specific order of the adjacency matrix is *m*×*m*(*m* = |*V*|), and the new topological connection of the stealthy complex network can be obtained after fusion as follows:

$$\left\{\begin{array}{l} M_{uv}=1,u\in V,v\in V,\left(u,v\right)\in E\\ M_{uv}=0,u\in V,v\in V,\left(u,v\right)\notin E \end{array}\right.$$ (2)

In the formula, *M*_*uv*_ represents the adjacency matrix; *u* and *v* represent sections in the adjacency matrix, respectively.

According to whether the suspected attack source in the complex network of stealing type can be monitored [16], it is divided into two subsets, *S* ⊆ *V* and *S*′ ⊆ *V*. *S* and *S*′ represent two subsets of attack sources respectively. Monitoring node *S*′ cannot cover the required point set due to resource constraints, so monitoring node *S* is configured as the main monitoring node. The expression of *S* is:

$$S=\left\{s_{1},s_{2},\ldots ,s_{j},\ldots ,s_{k}\left|k\leq M_{uv}\right.\right\}$$ (3)

In the formula, *s*_*k*_ represents the *k* suspected attack source.

*N* is used to represent a subset of ordinary nodes, then the expression of *N* is:

$$\left\{\begin{array}{l} N=\left(n_{1},n_{2},\ldots ,n_{i},\ldots ,n_{t}\left|t\leq M_{uv}\right.\right)\\ N=V-S-S\prime \end{array}\right.$$ (4)

In the formula, *n*_*t*_ represents the *t* ordinary node.

For the common node *n*_*i*_, it is selected as the monitoring node, then *n*_*i*_ needs the controller to carry out more stealth-type complex network information interaction, and occupy the bandwidth resources of some links.

#### 2.2.2 Cost selection of a single node

The shortest distance from *n*_*i*_ to the controller is denoted as [*i*, *c*], where *i* = 1, 2, …, *t* and the number of hops is denoted as *d*_*i*_; the shortest distance from *n*_*i*_ to the suspected attack source *s*_*ij*_ is denoted as, where *i* = 1, 2, …, *t*, *j* = 1, 2, …, a, and the number of hops is denoted as *d*_*ij*_.

The node cost is calculated by considering resource cost and communication cost [17]. Among them, the communication cost is:

$$C_{cn(\tau )}=d_{\tau }+\sum_{\upsilon }d_{\tau \upsilon }$$ (5)

In the formula, *C*_*cn*(*τ*)_ represents the communication cost, *τ* represents the number of the monitoring node, and *υ* represents the number of the suspected attack node in the monitoring range corresponding to the monitoring node.

The calculation formula of resource cost is as follows:

$$C_{s(\varphi )}=C_{sl}-\frac{C_{sl}-C_{sh}}{1+a\times e^{-b\times (B_{\left(\varphi \right)}-B_{l})}}$$ (6)

$$a=\frac{C_{sh}-C_{sl}}{\varepsilon }-1$$ (7)

$$b=\frac{2}{\left(B_{h}-B_{l}\right)}\times In\left(\frac{C_{sl}-C_{sh}-\varepsilon }{\varepsilon }\right)$$ (8)

In the formula, *φ* in resource cost refers to ordinary node number; *C*_*s*(*φ*)_ represents resource cost; *C*_*sl*_ represents the resource cost when the host load is 0; *C*_*sh*_ represents the resource cost corresponding to the maximum load of the host; *a* and *b* respectively represent the specific difference between the fitting threshold value of Logistic Curve [18] and the busyness degree of stealthy complex network equipment. The determination of the threshold depends on the specific application scenario and target. Under the maximum load of the host, the threshold is set to 0.9. When the host load is close to the maximum, measures are taken to avoid system performance degradation and service interruption caused by excessive load, and to balance system load and performance. *B*_(*φ*)_ represents the specific resource idle rate of common node *φ*; *B*_*l*_ stands for low equipment idle rate; *B*_*h*_ stands for high device idle rate; *ε* stands for minimal constant.

The calculation formula of the cost of a single node is as follows:

$$Cost_{\tau }=C_{cn(\kappa )}+C_{s(\varphi )}$$ (9)

In the formula, *C*_*cn*(*κ*)_ represents the cost coefficient of a single node.

#### 2.2.3 Monitoring node algorithm

For each suspected attack node *s*_*j*_, select *can*_*j*_ as the alternative monitoring node from *N* and put it into the alternative set of *Can*. At this point, the proportion of each suspected attack node in the node set is:

ϑ=i∑i≠jCan−Canj+sjN (10)

Make statistics on the specific occurrence times of each monitoring alternative node in *Can*, and select the node with the most occurrence times as the result of sequential selection [19]. Remove the nodes selected once and the suspected attack nodes covered by this node from the complex network of stealthy-type, and obtain the new average cost arrays of *M*_*uv*_, *S* and *AC* in the removed topology [20].

For *M*_*uv*_, *S* and *AC*, repeat the above steps until *S* = ∅ to complete the selection of nodes.

In the real complex network environment of stealthy type, node transformation in the complex network of stealthy type is fast and relatively random. Assuming that the initial position coordinates of the nodes to be monitored in the complex network of stealthy type are (*x*_*s*_, *y*_*s*_) and (*x*_*j*_, *y*_*j*_), then the extracted monitoring nodes are as follows:

$$L_{ij}=\sqrt{{\left(x_{s}-x_{j}\right)}^{2}+{\left(y_{s}-y_{j}\right)}^{2}}$$ (11)

In the formula, (*x*_*s*_ − *x*_*j*_)^2^ represents the predicted position of the estimated node and the actual position of (*y*_*s*_ − *y*_*j*_)^2^ node.

### 2.3 Stealing complex network attack monitoring

In the invisible complex network, the probability distribution of node transformation is calculated according to the characteristics and laws of node transformation, which is used as the reference value. Based on the selection of the above nodes, through real-time monitoring of Packet-In frequency messages, the stealthy complex network attacks in the monitoring node can be judged, and the stealthy complex network attacks can be monitored. Monitoring Packet-In frequency messages requires judging the frequency of their messages and updating their reference datasets [21].

Gaussian distribution is used to fit the message frequency data. The fitting formula is as follows:

$$p\left(x\right)=\frac{1}{\sqrt{2\pi }\sigma }•e^{-\frac{{\left(x-\mu \right)}^{2}}{2\times \sigma^{2}}}$$ (12)

In the formula, *p*(*x*) represents the Gaussian distribution, *σ* represents the standard deviation corresponding to the Gaussian distribution, *μ* represents the mean value of the Gaussian distribution, and *x* represents the message frequency data.

**Fig 2:**
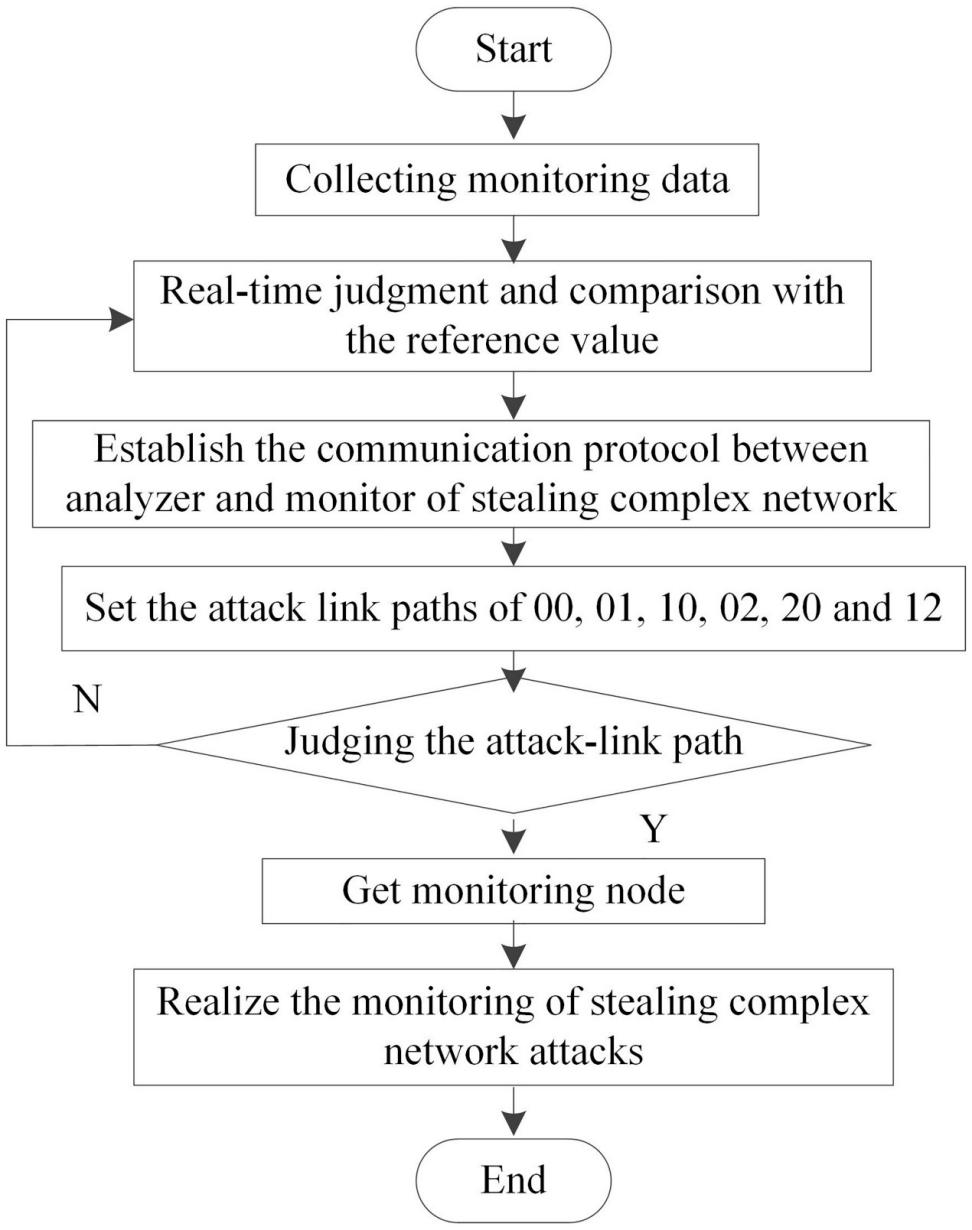
Overall flow chart of monitoring node algorithm.

The collected monitoring data are substituted into the above formula for calculation, and the specific occurrence probability of the current time and frequency is obtained, which is compared with the reference value for judgment in real time. The oldest information is removed from the training data set, and the data collected in the current period is added by referring to the data set. The AS (Application Server) level path is regarded as part of the attack path when tracing the inter-domain attack source. It is also necessary to construct a communication protocol between analyzers and between analyzers and stealing complex network monitors to realize the sharing of attack information. The communication protocol is obtained on the basis of extended IP (Internet Protocol) packets, and the information area and message flags are added in the IP options. Among them, there are six types of attack link information, including 00, 01, 10, 02, 20 and 12. 00 denotes a hidden complex network monitor-analyzer. This attack link is that the attack source sends an attack path request message to the analyzer through the hidden complex network monitor. 01 denotes a perimeter analyzer-a hidden complex network monitor. This attack link is that an attack source sends an attack path request message to the hidden complex network monitor through the perimeter analyzer. 10 denotes a hidden complex network monitor-a hidden complex network monitor. This attack link is that an attack source sends a query path request message through communication between hidden complex network monitors. 02 stands for Surround Analyzer-Surround Analyzer. This attack link is that the attack source sends the attack path request message through the communication between the surrounding analyzers. 20 denotes a hidden complex network monitor-non-analyzer, and this attack link is that the attack source sends a query path request message to the non-analyzer through the hidden complex network monitor. Reference numeral 12 denotes a perimeter analyzer-a non-covert complex network monitor. This attack link is that an attack source sends an attack path request message to the non-covert complex network monitor through the perimeter analyzer. By judging the attack-link attack path, the monitoring node algorithm is designed. The overall flow of the monitoring node algorithm is shown in Fig 2.

## 3 Experimental analysis

### 3.1 Experimental environment

**Fig 3:**
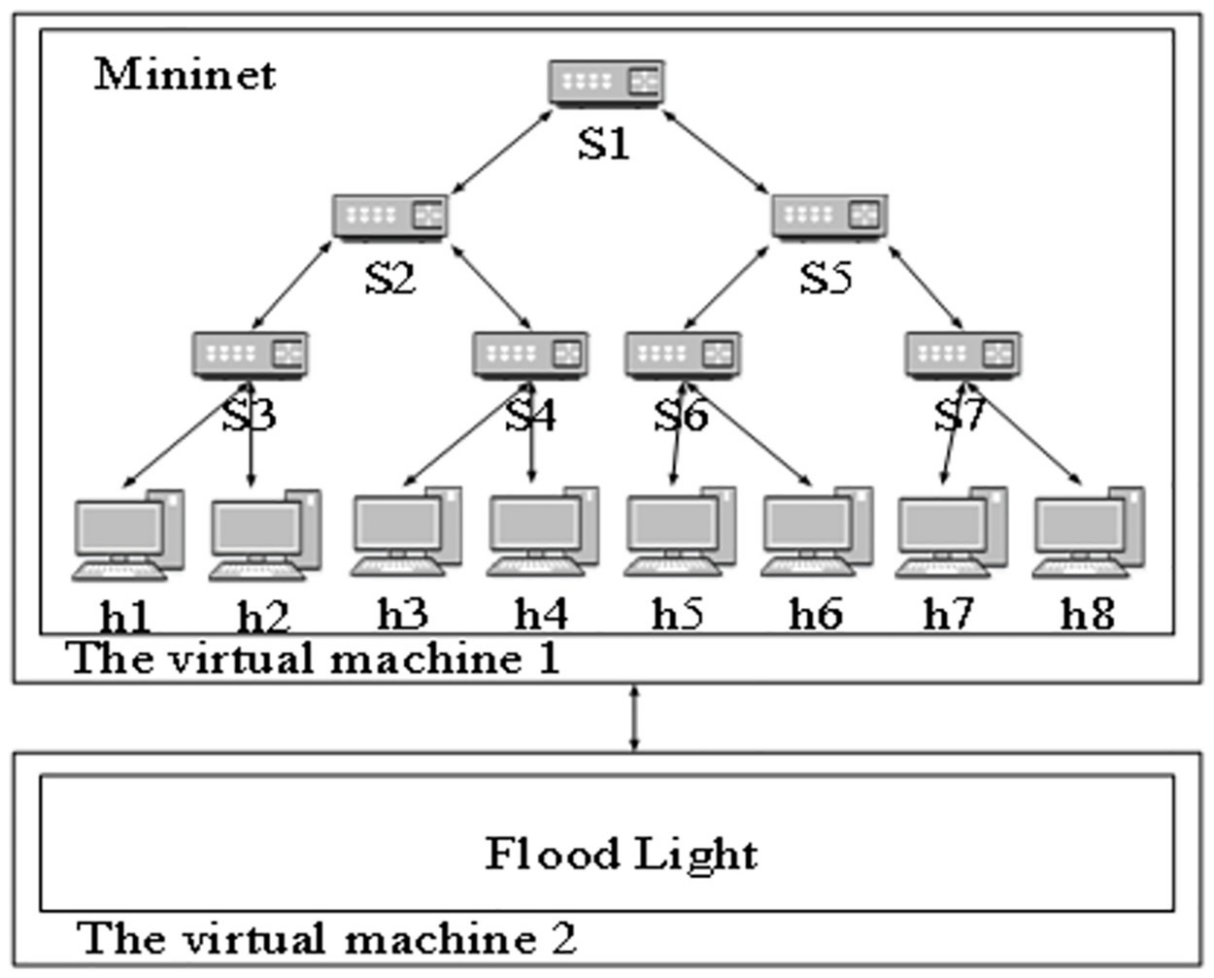
Topology structure of stealing complex network in simulation experiment domain.

In order to verify the effectiveness of the proposed method, simulation experiments are carried out. Two virtual machines, Floodlight and Mininet, are used to build a complex network topology, including forwarding links, hosts and devices, and Floodlight is used to control the remote connection of Mininet and control and manage the whole experimental domain. The topology of the stealing complex network in the simulation experiment domain is shown in Fig 3.

Combined with Visual C + and Matlab R2010, the design of stealthy complex network attack monitoring is carried out. In the training process, the characteristic components of stealth complex network attack monitoring are extracted from the security situation awareness information read by using simulation tools, and the specific steps are as follows:

1. Normalize and standardize the extracted features, and set the distribution of the edge contour feature set of the stealth complex network attack monitoring information to 0.12 to enhance the performance of the model.
2. According to the data characteristics, the matching coefficient of the regional block template of the monitoring information is set to 0.27, the gray pixel set size of the monitoring information is 12, and the edge pixels are set to 0.6.
3. Using random gradient descent to compare the error between the predicted output of the network and the actual target output, and update the parameters. Finally, the learning iterations of stealth complex network attack monitoring are set to 120 times, and the simulation step size is 20 times.
4. According to the above simulation environment and parameters, the monitoring method of stealth complex network attack is simulated and analyzed.

### 3.2 Experimental parameters

The specific device parameter information in the simulation experiment domain is shown in Table 1.

**Table 1:** Device parameter information in simulation experiment domain.

Serial number of equipment	MAC address	IP address
h1	9a:1d:3c:ec:d1:4c	10.1.1.1
h2	67:a6:8e:05:3d:1e	10.1.1.2
h3	65:51:6a:10:4b:1f	10.1.1.3
h4	23:78:de:e8:c8:87	10.1.1.4
h5	26:15:90:36:0d:8b	10.1.1.5
h6	7e:d7:73:03:08:04	10.1.1.6
h7	E1:a1:c6:be:a1:f4	10.1.1.7
h8	ee:e4:37:48:5c:1d	10.1.1.8

Run the attack script in host H1 to generate stealing complex network attack data stream. In the experiment, the data stream of stealing complex network attack is ICMP message. In the process of monitoring complex stealthy network attacks, 1000 to 4000 nodes in the complex stealthy network were obtained and monitored. Resource occupancy rate and monitoring accuracy were used as indicators. When the unattacked node is marked as 0, the same data will be searched in the attack tree. When the node is marked as 1, all other nodes with the same address information will be changed to 2. The attack path of the stealthy complex network is constructed by searching the attack tree of the original stealthy complex network, and the propagation path of the attack is traced back. In the attack tree of original stealthy complex network, the attack path of stealthy complex network is determined and highly suspected by association analysis and inference, and the unattacked nodes are pruned.

### 3.3 Analysis of results

#### 3.3.1 Analysis of resource occupancy rate in monitoring complex stealthy network attacks

**Fig 4:**
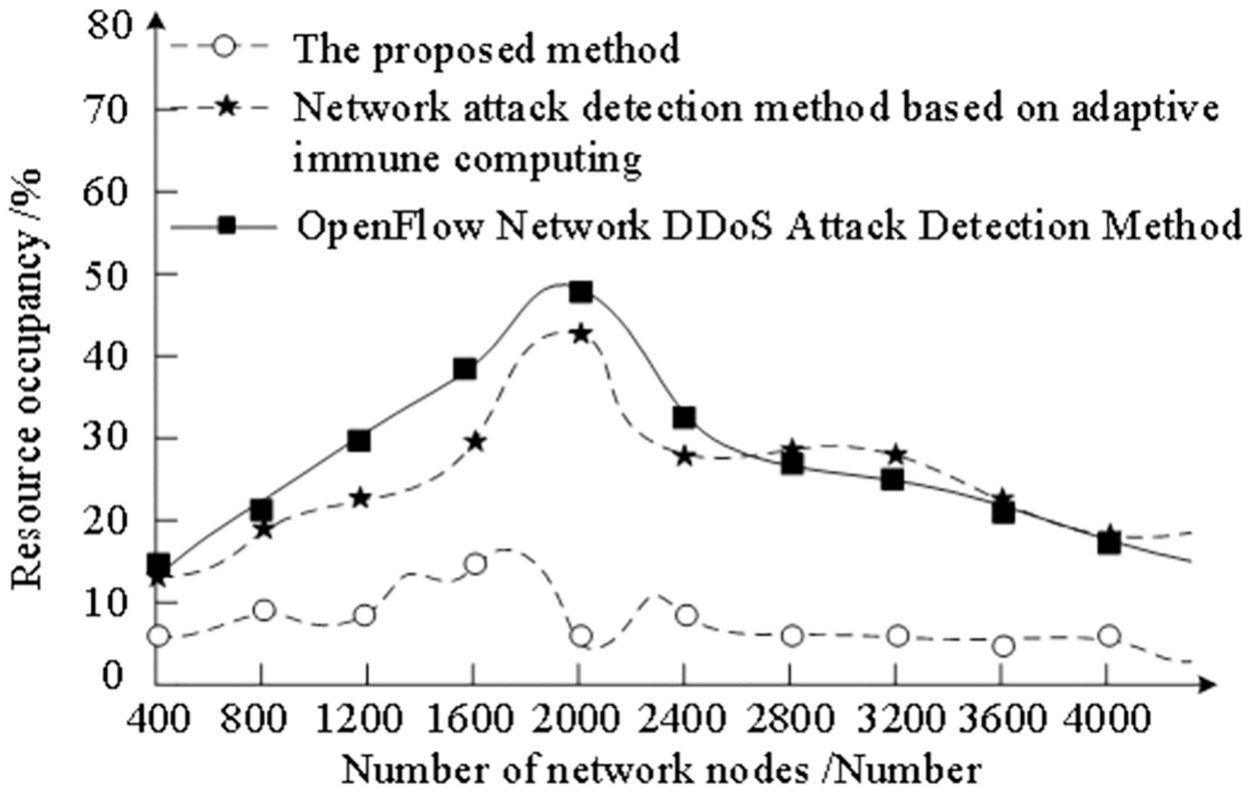
Analysis of resource occupancy rate for stealing complex network attack monitoring.

In order to verify the effectiveness of the proposed method, the experiment analyzes the resource occupancy rate of the proposed method, the detection of stealing complex network attacks based on adaptive immune computation and the detection of OpenFlow DDoS attacks. The experiment results are shown in Fig 4.

The analysis of Fig 3 shows that the resource occupancy rates of the three methods are different with the change of the number of nodes in a complex stealthy network. Among them, the resource occupancy rate of the proposed method is about 15%, while the resource occupancy rate of the other two methods is always higher than the proposed method. This is due to the security situation awareness of datagram information, the construction of traceability model of stealing complex network attack, the effective analysis of stealing complex network security situation, and the selection of monitoring nodes by calculating the cost of monitoring nodes, thus reducing the occupancy rate of monitoring resources, which proves that the proposed method has some advantages.

#### 3.3.2 Accuracy analysis of different methods for monitoring stealing complex network attacks

**Fig 5:**
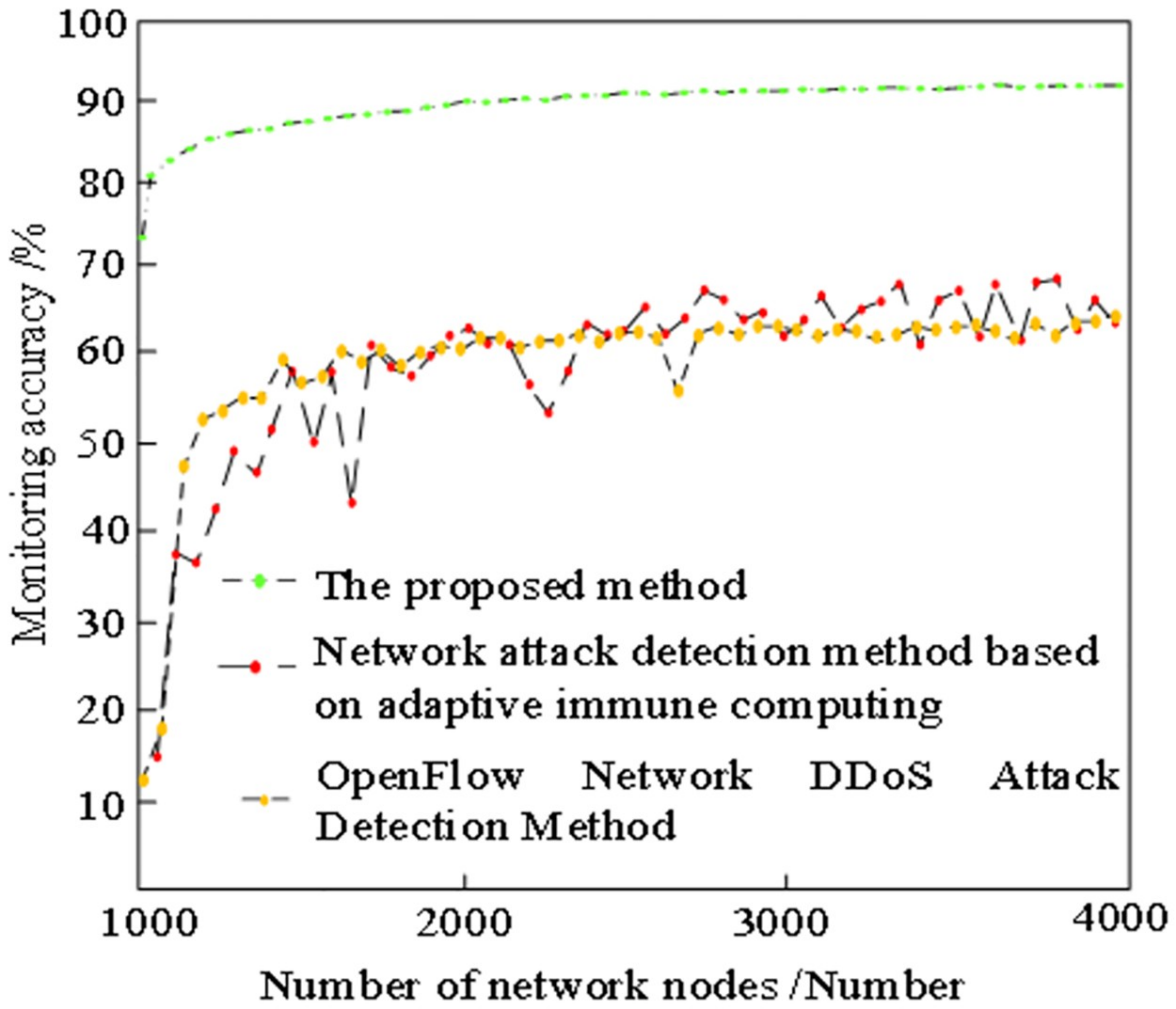
Comparison of monitoring accuracy of different stealing methods for complex networks.

To further verify the feasibility of the proposed method, experiments are conducted to analyze the accuracy of the proposed method, the detection of stealing complex network attacks based on adaptive immune computation and the detection of sample nodes by the OpenFlow DDoS attack detection method. The experimental results are shown in Fig 5.

Analysis of Fig 5 shows that under the same experimental conditions, there are some differences in the accuracy of the three methods. Among them, the monitoring accuracy of the proposed method is high, up to about 92%, while the monitoring accuracy of other methods is lower than the proposed method. This is because this method can identify and track the attack behavior more accurately by constructing the tracking model of invisible complex network attacks. This model can deeply analyze the network traffic and behavior patterns, so as to find abnormal and suspicious activities, and effectively improve the monitoring accuracy. At the same time, this method selects public monitoring nodes for monitoring, which play an important role in location and information transmission in the network. By monitoring these nodes, the security situation of the network can be obtained and judged based on the attack behavior. The cost of a single monitoring node is calculated by the algorithm, and the monitoring node is determined by the monitoring node algorithm. This algorithm can comprehensively consider the importance, cost and characteristics of attack behavior of nodes, select the most representative monitoring nodes, further improve the accuracy of monitoring, and effectively identify and defend attacks in complex network environment.

#### 3.3.3 Analysis of training time of different methods for monitoring stealing complex network attacks

**Fig 6:**
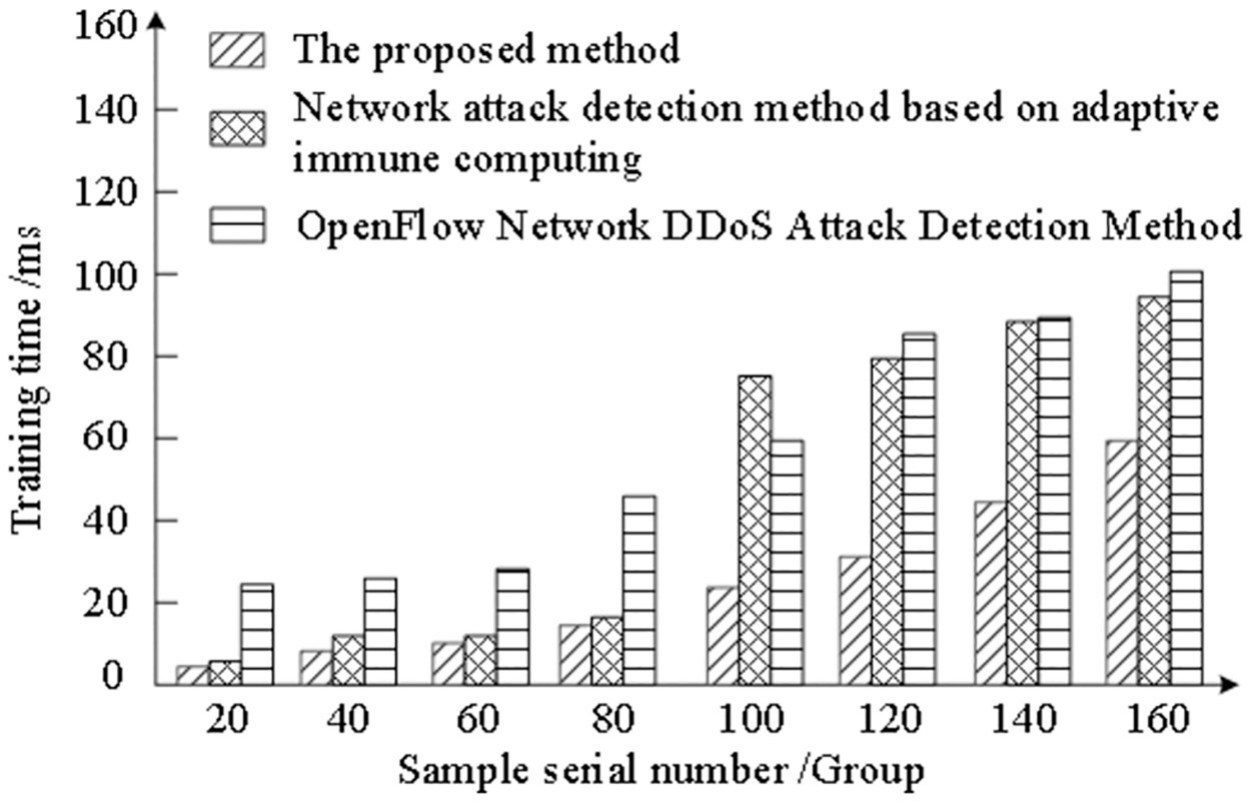
Analysis of training time of different methods for monitoring stealing complex network attacks.

Different training time will affect the performance of the monitoring method, and the shorter the training time, the better the response efficiency of the system. 160 groups of network attack data are selected as samples. After data cleaning, feature extraction and label classification, the proposed method, network attack detection method based on adaptive immune computing and OpenFlow network DDoS attack detection method are used to train the models respectively, and the training time of each method is recorded. The experiment results are shown in Fig 6.

By analyzing Fig 6, it can be seen that the training time of the proposed method, the network attack detection method based on adaptive immune computing and the OpenFlow network DDoS attack detection method is increasing with the increase of the number of samples. However, by comparing the test results of the above methods, it is found that the training time of the proposed method is shorter than that of the network attack detection method based on adaptive immune computing and the OpenFlow network DDoS attack detection method. When training 160 sets of data, the proposed method can complete the training in 60ms, while the network attack detection method based on adaptive immune computing and the OpenFlow network DDoS attack detection method need to complete the training in 95ms and 100 ms. Moreover, the network attack detection method based on adaptive immune computing has experienced a sharp increase in training time when training 100 groups. This shows that the proposed method has high system response efficiency, because the proposed method uses Gaussian distribution to fit the message frequency data and establishes a communication protocol to complete the sharing of attack information, thus improving the training time and the system response efficiency.

## 4 Conclusion

In this study, the tracking model of stealth complex network attacks is constructed, and the monitoring nodes of stealth complex network attacks are determined by using the monitoring node algorithm, which effectively reduces the resource occupancy rate of monitoring nodes and improves the monitoring accuracy. This research result provides important reference and guidance for the current research and practice in the field of security situation awareness. In view of the ever-changing network environment and attack means, we will continue to improve the monitoring method of security situation-aware stealing complex network attacks on the basis of the research results to ensure the continuous improvement of network security protection level. At the same time, strengthen interdisciplinary cooperation with related fields, constantly promote the development of security network monitoring technology by using the latest technologies and methods, and contribute to building a safer and more stable network environment to adapt to the increasingly complex and changeable network security challenges. Therefore, the research results of the monitoring method of stealing complex network attacks considering security situation awareness not only provide new ideas and methods for current network defense, but also lay a solid foundation for future network security research.
